# Supplementary material for: Mechanisms governing the pioneering and redistribution capabilities of the non-classical pioneer PU.1
Source: Nat Commun. 2020 Jan 21;11:402. doi: 10.1038/s41467-019-13960-2 (PMC6972792; doi:10.1038/s41467-019-13960-2)
Supplement: Supplementary file 7 — Source data [file 41467_2019_13960_MOESM7_ESM.zip › Source_Data/Figure5/Figure5A_MotifScanOutput/homerResults/motif3.similar.html]

motif3

## Information for motif3

T
A
G
C
G
C
T
A
A
T
G
C
G
C
T
A
C
G
A
T
T
A
G
C
C
G
T
A
T
G
C
A
C
G
T
A
T
A
C
G
T
A
C
G
  
Reverse Opposite:  

A
T
G
C
A
T
G
C
A
C
G
T
A
C
G
T
A
C
G
T
A
T
C
G
C
G
T
A
C
G
A
T
T
A
C
G
C
G
A
T
A
T
C
G
  

|  |  |
| --- | --- |
| p-value: | 1e-146 |
| log p-value: | -3.382e+02 |
| Information Content per bp: | 1.609 |
| Number of Target Sequences with motif | 1301.0 |
| Percentage of Target Sequences with motif | 43.14% |
| Number of Background Sequences with motif | 9718.0 |
| Percentage of Background Sequences with motif | 21.30% |
| Average Position of motif in Targets | 228.5 +/- 163.9bp |
| Average Position of motif in Background | 209.5 +/- 136.7bp |
| Strand Bias (log2 ratio + to - strand density) | 0.0 |
| Multiplicity (# of sites on avg that occur together) | 1.33 |
| Motif File: | file (matrix) reverse opposite |

### Similar de novo motifs found

|  |  |  |  |  |  |  |  |
| --- | --- | --- | --- | --- | --- | --- | --- |
| Rank | Match Score | Redundant Motif | P-value | log P-value | % of Targets | % of Background | Motif file |
| 1 | 0.960 | C G T A T A G C G C T A C G A T A G T C C G T A T C G A T C G A T A C G | 1e-131 | -303.494786 | 37.53% | 17.88% | motif file (matrix) |
| 2 | 0.969 | A T G C C A G T A C G T C G A T A T C G C G T A C G A T T A C G C A G T A T C G | 1e-130 | -300.968946 | 37.80% | 18.16% | motif file (matrix) |
| 3 | 0.940 | A C G T A T C G A G T C A C G T A G C T A C G T A C T G G C T A C G T A T A C G G C A T A T C G | 1e-112 | -259.760753 | 15.78% | 4.43% | motif file (matrix) |
| 4 | 0.786 | A G T C A C G T A C G T A C G T A T G C C G T A C G A T A T C G | 1e-91 | -211.493870 | 22.55% | 9.46% | motif file (matrix) |
| 5 | 0.893 | A G T C A C G T A C G T A C G T A T C G C G T A C G T A | 1e-75 | -174.261441 | 38.59% | 23.00% | motif file (matrix) |
| 6 | 0.792 | C A T G A T G C C G A T A G C T C G A T A T C G G C T A C T G A T A C G C G A T A C T G C T A G C A G T | 1e-65 | -150.308995 | 8.55% | 2.22% | motif file (matrix) |
| 7 | 0.761 | C G T A A G T C C G T A A C G T A G T C C G T A C G T A | 1e-43 | -99.057803 | 19.43% | 10.56% | motif file (matrix) |
| 8 | 0.798 | G C A T G T A C C G A T C A G T G T A C C G T A G T C A T C G A C A T G A T G C G A C T A G T C G T A C | 1e-39 | -90.011820 | 27.39% | 17.36% | motif file (matrix) |
| 9 | 0.770 | A C T G A C G T A C G T A C G T A C T G C G T A A C G T A G T C | 1e-27 | -64.160774 | 3.28% | 0.77% | motif file (matrix) |
